# Cross-cultural adaptation and validation of the Swedish version of the Modified Dental Anxiety Scale

**DOI:** 10.2340/aos.v83.42436

**Published:** 2024-12-16

**Authors:** Markus Höglund, Emma Göranson, Inger Wårdh, Pernilla Larsson

**Affiliations:** a Center for Orofacial Medicine, Public Dental Service Östergötland, Linköping, Sweden; b Department of Dental Medicine, Karolinska Institutet, Huddinge, Sweden; c Center for Orthodontics and Pediatric Dentistry, Norrköping, Public Dental Service Östergötland, Sweden; d Department of Orthodontics, Malmö University, Malmö, Sweden; e Academic Centre for Geriatric Dentistry, Stockholm, Sweden; f Department of Health Sciences, Karlstad University, Karlstad, Sweden; g Centre for Oral Rehabilitation, Folktandvården Östergötland, Norrköping, Sweden; h Department of Prosthetic Dentistry, Malmö University, Malmö, Sweden

**Keywords:** Dental anxiety, dental fear, screening instrument, validation

## Abstract

**Introduction:**

The impact of dental anxiety is profound. At the same time, dental anxiety is sometimes difficult to detect. Therefore, a patient-reported outcome measure is needed. The Modified Dental Anxiety Scale (MDAS) is a short, internationally used self-assessment questionnaire for screening of dental anxiety.

**Aim:**

To cross-culturally adapt the original English MDAS to Swedish (MDAS-S), and to validate it in a Swedish setting.

**Materials and methods:**

The adaptation was conducted in accordance with recommended guidelines. Field testing was performed both among adults presenting for their regular dental check-ups and among adults diagnosed by a psychologist as dentally phobic.

**Results:**

The MDAS-S was formed during the adaptation procedure. Field testing included 246 adults presenting for their regular dental check-ups and 7 adults diagnosed with dental phobia. The MDAS-S score was significantly higher (*p* < 0.001) in the dentally phobic group than in the regular dental check-up group. Reliability was good with Cronbach’s Alpha values between 0.880 to 0.909. Test-retest of 37 individuals showed an excellent Intraclass Correlation Coefficient of 0.956. Confirmatory factor analysis (CFA) finds support for a two-factor model although with 78% shared variance between the factors.

**Conclusions:**

The MDAS-S demonstrates good reliability and appears valid as a screening tool for dental anxiety among Swedish adults.

## Introduction

Dental anxiety is a commonly encountered phenomenon that affects a significant portion of the population [1], most frequently among women and younger adults [2]. A patient’s level of dental anxiety varies on a continuous scale from none to extreme, and its most severe form can be considered a specific phobia [3]. Dental anxiety is associated with untreated dental decay and missing teeth [4, 5]. It is also associated with avoidance of routine dental care and increased frequencies of emergency treatments, usually motivated by pain [6]. Furthermore, dental anxiety is correlated with low confidence in handling dental anxiety [7] and poor Oral Health-Related Quality of Life [8].

Several techniques exist to manage and treat dental anxiety [9, 10]. To choose the right technique, a dental clinician must first identify a patient as dentally anxious and then assess the patient’s level of dental anxiety. However, several studies have found that dental clinicians face difficulties with assessing a patient’s level of dental anxiety [7, 11–13]. Thus, a short self-rating questionnaire like Modified Dental Anxiety Scale (MDAS) is a suitable screening tool to help dental clinicians with this task [14].

The MDAS, developed in the 1990s by Humphris et al. [15], is among the most frequently used self-rating questionnaires used in a scientific setting [14]. The MDAS consists of five questions. In each question, the subjects are asked to imagine that they will soon be exposed to five different dental procedures and to rate how anxious they would feel in these hypothetical situations. Each question is answered using the same five-point Likert rating scale, with answers ranging from ‘not anxious’ to ‘extremely anxious’. Answering the MDAS does not increase the patient’s level of dental anxiety [16]. Patient’s report lower dental anxiety if they believe that the dental clinicians know that they suffer from dental anxiety [17]. The MDAS has primarily been seen as a one dimensional scale measuring dental anxiety. Growing evidence suggests a two dimensional model with two questions measuring anticipatory dental anxiety and three questions measuring treatment related dental anxiety [18, 19]. The MDAS is simple to use and has been cross-culturally adapted and validated in numerous languages [20–23]. It has shown good cross-cultural reliability and validity [24] but has yet to be cross-culturally adapted and validated for a Swedish setting.

### Aim

This study aims to cross-culturally adapt and validate a Swedish version of the MDAS

## Materials and methods

### Formation of the MDAS-S

The adaptation of the MDAS to the Swedish version (MDAS-S) was performed following the Guidelines for Translation and Cultural Equivalency of Instruments established by the International Research Diagnostic Criteria for Temporomandibular Disorders (RDC/TMD) Consortium [25]. These guidelines are a synthesis of multiple recommendations across several disciplines. The adaptation was coordinated and supervised by a team leader (first author). All translators as well as the reviewer were blinded to all except their own steps of the translation. The measurement properties of the MDAS-S were assessed according to Consensus-based Standards for the selection of health Measurement Instruments (COSMIN) [26] methodology.

The cross-cultural adaptation and validation followed the following process:

#### Forward translation

Forward translation of the MDAS was performed independently by two forward translators, and after discussion this translation was synthesised into a first draft. Both forward translators had Swedish as their native language and were fluent in English. One of the forward translators was a dentist experienced in treating patients with dental anxiety, and the other a lay person naive to the instrument’s intent.

#### Back translation

The first draft of the MDAS-S was translated back into English by the back translator who was a lay person with English as his native language and Swedish as a fluent secondary language.

#### Independent review

The back translation of the MDAS-S was reviewed by one of the original MDAS creators and compared to the original MDAS for any significant discrepancies.

#### Repeating cycle of translation

The forward translators were informed of any significant discrepancies, prompting a new cycle of forward translation, back translation, and review. When no significant discrepancies could be found between the original MDAS and the back translations, the translation cycle ended and the preliminary MDAS-S was established.

#### Expert panel review

The face validity of the preliminary MDAS-S was then evaluated by two focus groups, the first consisting of five experienced dentists and the second of five lay persons. Based on their recommendations, the authors revised the preliminary MDAS-S, establishing the final MDAS-S. By doing so, the authors put great effort into not creating any new significant discrepancies between the MDAS and the MDAS-S.

### Field testing

Test subjects from two different settings were recruited for the study. The samples were chosen in order to recruit groups with and without dental phobia to allow for analysis of discriminant validity.

The first setting was adult dental patients presenting for their regular dental check-up. These were recruited from four public dental clinics in Östergötland, Sweden; and from two private dental practices located in Linköping and Stockholm. These test subjects were approached for possible recruitment in the waiting room prior to treatment. The larger public dental clinics were asked to stop recruiting when they reached 50 participants each and the smaller private practices were to stop when they reached 25 participants each (in total 250 participants).

The second setting was dentally phobic individuals recruited among adults seeking help from a psychologist in Linköping for severe dental anxiety. These test subjects were diagnosed by a psychologist as dentally phobic according to the criteria for specific phobia according to the Diagnostic and Statistical Manual of Mental Disorders, Fifth Edition (DSM-V) [27]. They were recruited on their first visit to the psychologist. The psychologist were instructed to stop after recruiting seven participants.

Verbal and written information about the study was given to all test subjects in both settings and they had the opportunity to ask questions prior to signing a written consent. All test subjects answered the same questionnaire containing the following parts: (1) The newly adapted MDAS-S, five questions regarding dental anxiety during different future imaginary dental procedures, answered on a five-point Likert scale, with a possible total score ranging from 5 to 25. (2) The Swedish version of the short form Oral Health Impact Profile (OHIP 14-S), containing 14 questions regarding oral health related quality of life, answered on a five-point Likert scale with a possible total score ranging from 0 to 52. (3) The following three single answer questions answered on 100 mm visual analogue scales (VAS), ‘How dentally anxious are you?’ assessing dental anxiety, ‘How do you deal with possible anxiety in connection with your dental care?’ assessing confidence in one’s ability to handle dental anxiety, and ‘How satisfied are you with your general health?’ assessing general health. (4) Finally, the questionnaire contained two questions regarding sex and age.

The test-retest procedure was performed with help from 50 subjects from the private dental practices. They were instructed to redo the MDAS-S at their convenience after approximately 2 weeks from their dental visit and then send it to the first author in a prepaid envelope.

#### Reliability

Internal consistency was tested with Cronbach’s Alpha and Cronbach’s ‘Alpha if item deleted’, Alpha values of 0.70 were considered adequate [28]. Test-retest was tested with Intraclass Correlation Coefficient (ICC) with a two-way random effects and consistency model, values less than 0.5 indicates poor reliability, between 0.5 and 0.75 moderate reliability, between 0.75 and 0.9 good reliability, and greater than 0.90 excellent reliability [29].

#### Validity

Face validity was assessed in the expert panel review. Discriminative validity was assessed by comparing MDAS-S scores between patients diagnosed with dental phobia and patients returning for a regular annual check-up, assuming a significant difference between the groups. Convergent validity was primarily assessed by correlating the MDAS-S with another self-rating scale for dental anxiety (VAS-DA) assuming a strong correlation between the scales. Convergent validity was further tested by correlating the MDAS-S with five factors known to associate with dental anxiety; female sex [2], young age [2], low self-confidence in handling dental anxiety (VAS-Confidence) [7] ,and low level of satisfaction with one’s general and oral health (VAS-Health and OHIP-14) [8]. Assuming a significant difference in MDAS-S scores between males and females as well as a moderate to weak correlation between the MDAS-S and the other four above mentioned factors associated with dental anxiety. Cross-cultural validity was assessed by comparing the results of the MDAS-S score to other published results of the MDAS in similar populations.

### Statistics

IBM SPSS version 27 and IBM AMOS were used for all statistical analyses, and the level of significance was set to 5%. Descriptive statistics, the Shapiro–Wilk test of normality, ICC, Cronbach’s Alpha, Cronbach’s Alpha ‘if item deleted’, Spearman’s rho, the Mann-Whitney U Test, and Confirmatory Factor Analysis (CFA) were used. If any part of the questionnaire was left unanswered or partially unanswered, that part was excluded; the remaining parts were recorded and used for statistical calculations.

#### Confirmatory factor analysis

A one-dimensional model was compared to the two-dimensional model suggested by Yuan et al. [19]. Diagonally weighted least squares (DWLS) was used as estimator since the data were endogenous and categorical. The fit indices used were normed chi-square (X^2^/df), Root Mean Square Error of Approximation (RMSEA), Robust Comparative Fit Index (CFI), Robust Tucker Lewis Index (TLI), Akaike’s Information Criterion (AIC), and Bayesian Information Criterion (BIC).

#### Sample size calculation

Power calculations were performed prior to the study start. An Alpha of 0.05 and a Power of 80% were decided. Based on data from an earlier similar study [7], it was estimated that a minimum of 155 test subjects were required. It was estimated that a minimum of five dentally phobic test subjects were required for the Mann-Whitney U Test.

### Ethical approval

Ethical approval for the study was obtained from the Swedish Ethical Review Authority before the start of the study (Dnr 2021-06247-01).

## Results

It took four cycles of translation to establish the preliminary MDAS-S. The expert panel review suggested only minor language adjustments and concluded that the scale had high face validity.

### Descriptive data

A total of 253 adults were recruited, 246 from the dental settings and 7 diagnosed with dental phobia. Their descriptive data are presented in Table 1.

**Table 1:** Descriptive statistics.

	*n*	Age Mean (± SD)	Gender	MDAS-S Median (min-max) IQR	VAS-DA Median (min-max) IQR	VAS-Confident Median (min-max) IQR	VAS-Health Median (min-max) IQR	OHIP-14-S Median (min-max) IQR
Dental patients*	246	39.2 (± 18.8)	141♀ 102♂	9 (5–25) 6	9 (0–100) 24	89 (0–100) 27	80 (6–100) 21	4 (0–50) 10
Dentally phobic	7	42 (± 8.9)	4♀ 3♂	22 (16–25) 3	86.5 (68–97) 13	31 (0–50) 43	42.5 (16–77) 38	32.5 (28–44) 12

MDAS-S: Swedish version of the Modified Dental Anxiety Scale; VAS-DA: Visual Analog Scale of self-assessed Dental Anxiety; VAS-Health: Visual Analog Scale of self-assessed general health; OHIP-14-S: The Swedish version of the short form Oral Health Impact Profile; SD: Standard deviation; IQR: interquartile range.

* Adults presenting for a regular dental check-up.

### Test of normality

All variables were highly skewed, indicating a non-normal distribution

### Reliability

Internal consistency; MDAS-S had a Cronbach’s Alpha of 0.909 and the Cronbach’s Alpha ‘if item deleted’ ranged between 0.880 and 0.909, indicating good internal consistency.

Of the 50 recruited subjects, 37 (74%) who were asked to perform the retest returned their answers. The test-retest of the MDAS-S showed an average ICC of 0.956, indicating excellent reliability.

### Validity

The MDAS-S was strongly correlated with VAS-DA and low patient confidence (VAS-confidence). Further, the MDAS-S hade a low, but statistically significant, correlation with young age as well as low self-rated general and oral health (see Table 2).

**Table 2:** Correlations between MDAS-S, VAS-DA, Age, VAS-Confidence, VAS-Health and OHIP-14-S.

Spearman’s rho	VAS-DA	Age	VAS-confidence	VAS-Health	OHIP-14
MDAS-S	0.82*	−0.25*	−0.68*	−0.29*	0.28*
VAS-DA	-	−004^α^	−0.72*	−0.25*	0.27*

MDAS-S: Swedish version of the Modified Dental Anxiety Scale; VAS: Visual Analog scale; DA: Dental Anxiety; OHIP-14: The Swedish version of the short form Oral Health Impact Profile.

* Correlation is significant at the 0.01 level (2-tailed).

α Correlation is non-significant (*p* = 0.54).

The Mann-Whitney U Test revealed significant differences in MDAS-S scores between the dentally phobic group compared to adults presenting for their regular dental check-ups (*p* < 0.001) as well as significantly higher MDAS-S scores among females compared to males (*p* < 0.001).

### Confirmatory factor analysis

**Figure 1:**
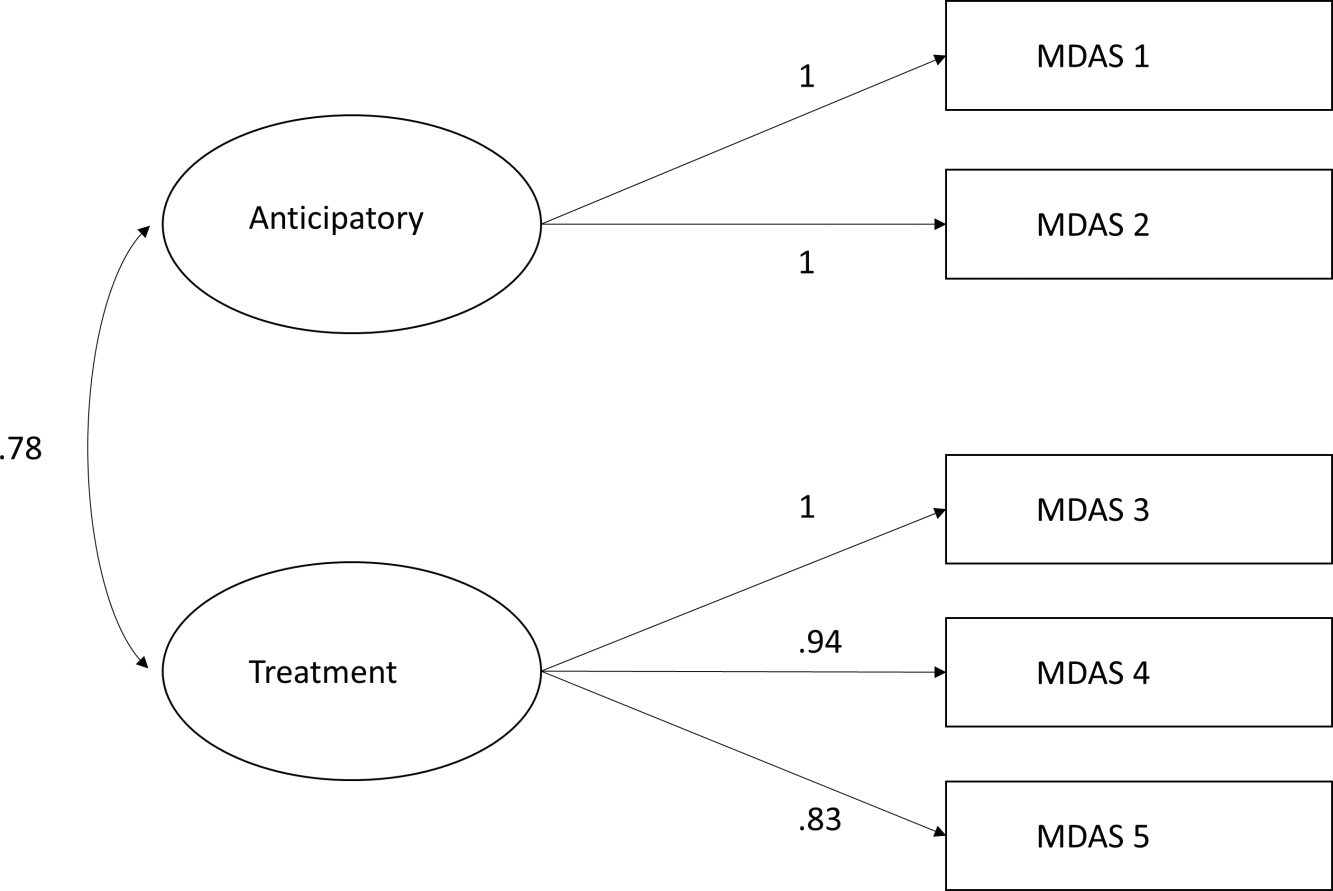
Factor structure loading of the two-dimensional model suggested by Yuan et al. [19] using diagonally weighted least squares estimation.

Table 3 presents multiple fit indices comparing the fit of the one and two dimensional model. All indices indicate that the two-dimensional model suggested by Yuan et al. [19] presents the best fit (see Figure 1).

**Table 3:** Confirmative factor analysis^α^ comparing the fits of the one and two dimensional model using multiple fit indices.

	X^2^	df	X^2^/df	RMSEA	CFI	TLI	AIC	BIC
One dimensional model	105.91*	5	21.12	0.17	0.88	0.76	2,785	2,838
Two dimensional model^β^	20.35*	4	5.09	0.03	0.99	0.99	2,702	2,758

X^2^: Chi-Square; df: degrees of freedom; X^2^/df: normed chi-square; RMSEA: Root Mean Square Error of Approximation; CFI: Robust comparative Fit Index; TLI: Robust Tucker Lewis Index; AIC: Akaike’s Information Criterion; BIC: Bayesian Information Criterion.

α using diagonally weighted least squares estimation,

β two-dimensional model suggested by Yuan et al. [19],

* *p* < 0.001.

The difference in chi-square between the one and two factor model was statistically significant (*p* < 0.0001).

### Cross-cultural validity

The mean MDAS-S score was 9.85, which is close to the result of the original MDAS, UK, general public population norm’s mean of 10.39 [20] and similar to but slightly lower than several other cross-culturally adaptations of MDAS [19, 21, 23, 30, 31].

## Discussion

This study has successfully cross-culturally adapted and established the MDAS-S as a suitable instrument for screening of dental anxiety among Swedish adults. The results in the validity section indicates that the MDAS-S primarily measures dental anxiety. The CFA gives further support for the thesis that the MDAS is two dimensional, measuring both anticipatory and treatment-related dental anxiety; however, with a very high covariance.

The strong correlation between the MDAS-S, VAS-DA, the high face validity of the expert panel review as well as the discriminative ability of the MDAS-S to distinguish between the dentally phobic and normal population presents strong arguments for the validity of the MDAS-S. The validity is strengthened further by the correlations between the MDAS-S and the other factors known from previous studies to be associated with dental anxiety ; female sex, young age, low self-confidence to handle dental anxiety, and low level of satisfaction with one’s general and oral health [2, 7, 8]. Furthermore the MDAS-S demonstrates good to excellent reliability. All fit indices from the CFA indicates that the two dimensional model offers a better theoretical fit than the one dimensional model. Although, the 78% shared variance between the latent factors limits the clinical usefulness of this finding. The CFI and the TLI indicate a close fit, the RMSEA below 0.06 indicates a good fit [32], and the normed chi-square indicates a reasonably close fit for the two dimensional model comparable to the results of Lahti et al. [18].

The correlation between MDAS-S and low confidence is in line with Bandura’s self-efficacy theory [33] that involves low specific self-efficacy in specific phobia. Although an established theory in psychology [34], there is lack of research on this connection among adults with dental anxiety. The association is interesting and points to a need of further studies.

The MDAS is among the most widely used instruments in surveys as well as clinical studies [14]. There are several other instruments that have greater length and go into greater detail than the MDAS. However, the main reasons that the MDAS stands out as one of the most widely used instruments for measuring dental anxiety is its short length, reliability, excellent test-retest stability, and general ease of use.

The use of a systematic adaptation process for the relatively short MDAS might at first seem a little overambitious. However, to be able to compare scores of the MDAS-S with other MDAS scores from different languages and cultural settings it is imperative that the adaptation process is rigorous, transparent, and reliable.

The cross-cultural adaptation of the MDAS-S was initially deemed as relatively simple, given its limited size and the cultural similarities between Sweden and the UK. However, there were still some hurdles to overcome. The biggest challenge was to translate the word ‘anxious’ into Swedish. Although several similar words exist in the Swedish language, no alternative was initially found that adequately captured all nuances of being anxious. This was proved during the initial cycle of reviews of the back translation as several words were disqualified as they were more closely related to other English words such as ‘fear’ and ‘worry’. Finally, the Swedish word ‘ängslig’ was found to be the most relevant. During the final back translation, it was proved to have a close cultural match, carrying approximately the same meaning as the word ‘anxious’.

The MDAS-S is validated primarily in a population of regular dental patients. This strengthens the clinical usefulness of the MDAS-S as a screening instrument of dental anxiety in the dental setting.

The mean score of the MDAS-S was lower than in many other adaptations; however, this makes sense when considering that we chose adults presenting for their regular dental check-ups and not a random sample of the entire population like most other studies. Therefore, the difference in scores, at least to some extent, can be explained by the habit of dentally anxious patients to avoid regular dental care [6].

Most of the participants in this study scored very low on their MDAS-S, suggesting a floor effect. However, it is more likely that the low score is explained by the low level of dental anxiety in the Swedish population [1]. Perhaps it is even desirable that most non-anxious patients score low as this facilitates the identification of more dentally anxious patients.

This study has several limitations. It did not record how many people refused to participate in the study. This means that we cannot estimate whether this contributed to any bias in the material. The reason behind this was of practicality; all clinics participated on a voluntary basis and we therefore wanted to minimise their administrative burden. The responsiveness of MDAS-S was not assessed in this study as changes over any extended period of time were not measured.

The strong correlation between the MDAS-S, the VAS-DA, the large difference in MDAS-S values between the dentally phobic and regular patients, and the reasonably close fit of the two dimensional model, altogether indicate that the MDAS-S has good construct validity and probably measures dental anxiety and nothing else.

## Conclusions

The MDAS-S demonstrates good to excellent reliability and appears valid as a screening tool for dental anxiety among Swedish adults.

## Supplementary Material

Cross-cultural adaptation and validation of the Swedish version of the Modified Dental Anxiety Scale
