# Supplementary material for: Cross-cultural adaptation and validation of the Swedish version of the Modified Dental Anxiety Scale
Source: Acta Odontol Scand. 2024 Dec 16;83:42436. doi: 10.2340/aos.v83.42436 (PMC11707684; doi:10.2340/aos.v83.42436)
Supplement: Cross-cultural adaptation and validation of the Swedish version of the Modified Dental Anxiety Scale [file AOS-83-42436-s1.pdf]

Supplementary material has been published as submitted. It has not been copyedited or typeset by Acta Odontologica Scandinavica.

Modified Dental Anxiety Scale
Swedish Version
(MDAS-S)

Skala för självskattning av tandvårdsrädsla

Hur ängslig känner du dig vid tandläkarbesök?
Kryssa i det alternativ som passar dig bäst

1. Om du skulle gå till tandläkaren I MORGON, hur skulle du känna dig?

Inte	Lite	Ganska	Mycket	Extremt
Ängslig ☐	Ängslig ☐	Ängslig ☐	Ängslig ☐	Ängslig ☐

2. **Om du skulle sitta i VÄNTRUMMET (inför behandling), hur skulle du känna dig?**

Inte	Lite	Ganska	Mycket	Extremt
Ängslig ☐	Ängslig ☐	Ängslig ☐	Ängslig ☐	Ängslig ☐

3. Om tandläkaren strax skulle BORRA I EN TAND, hur skulle du känna dig?

Inte	Lite	Ganska	Mycket	Extremt
Ängslig ☐	Ängslig ☐	Ängslig ☐	Ängslig ☐	Ängslig ☐

4. Om du strax skulle få TANDSTEN BORTTAGEN och TÄNDERNA POLERADE, hur skulle du känna dig?

Inte	Lite	Ganska	Mycket	Extremt
Ängslig ☐	Ängslig ☐	Ängslig ☐	Ängslig ☐	Ängslig ☐

5. Om du strax skulle få en SPRUTA med BEDÖVNING i tandköttet ovanför en kindtand i överkäken, hur skulle du känna dig?

Inte	Lite	Ganska	Mycket	Extremt
Ängslig ☐	Ängslig ☐	Ängslig ☐	Ängslig ☐	Ängslig ☐

Instruktioner för poängsättning (Den här delen ska inte patienten se, tas bort i patientversionen)
Varje svar ger följande poäng:

Inte ängslig	=	1
Lite ängslig	=	2
Ganska ängslig	=	3
Mycket ängslig	=	4
Extremt ängslig	=	5

Summan av de fem frågorna ger totalpoängen, min är 5 och max är 25: Cut-off värde i det engelska originalet är 19 eller högre, vilket där indikerar en mycket tandvårdsrädd patient, möjligen fobiskt rädd.

Contact details:
Markus Höglund markus.hoglund@regionostergotland.se
Centrum för Orofacial medicin +4610-105 95 35
58185, Linköping, Sweden
